# Supplementary material for: Bile acids drive the newborn’s gut microbiota maturation
Source: Nat Commun. 2020 Jul 23;11:3692. doi: 10.1038/s41467-020-17183-8 (PMC7378201; doi:10.1038/s41467-020-17183-8)
Supplement: Supplementary file 1 — Supplementary Information [file 41467_2020_17183_MOESM1_ESM.pdf]

Supplementary Information

Bile acids drive the newborn's gut microbiota maturation

van Best et al. (2020)

Supplementary Figure 1

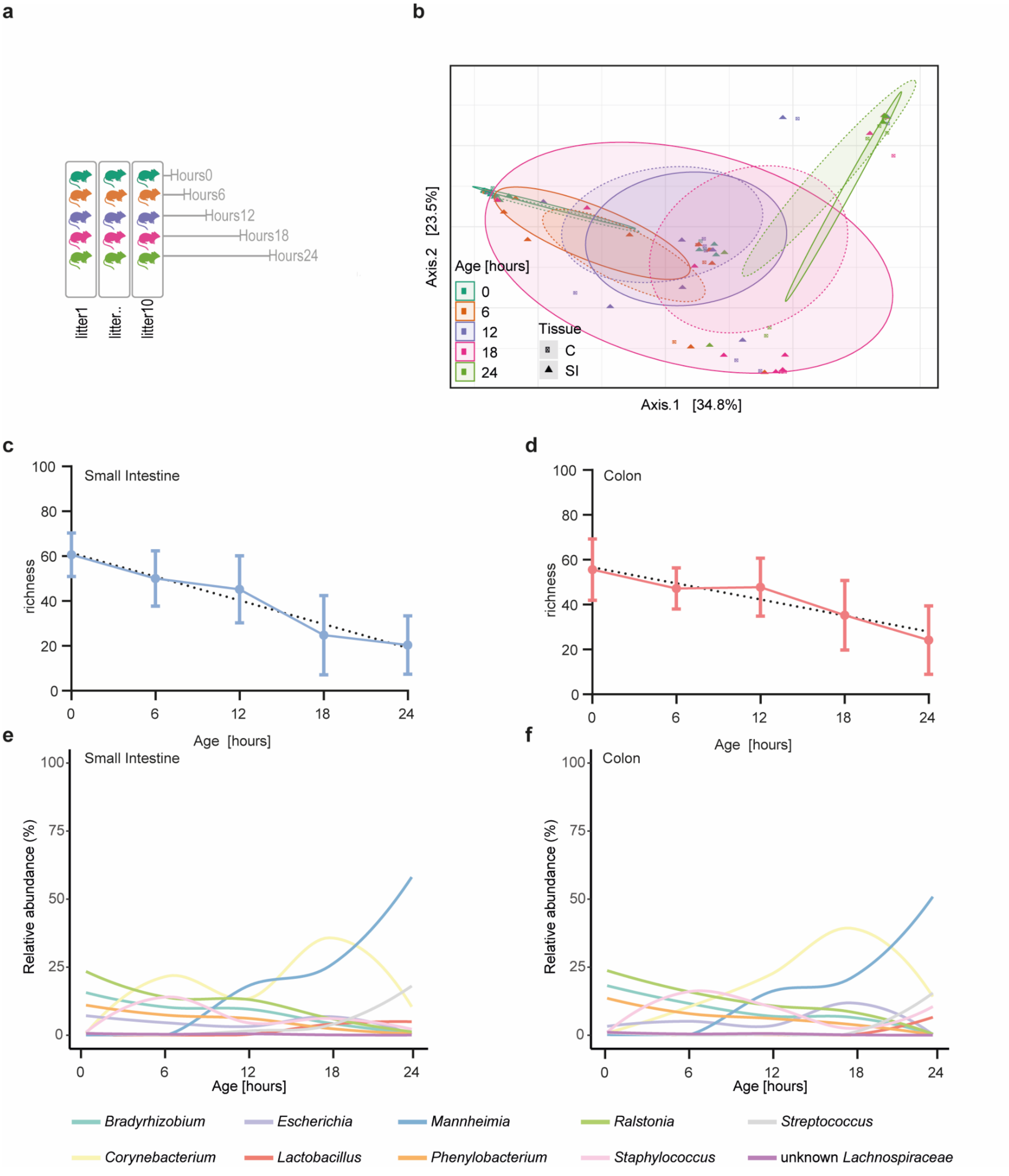

Supplementary Figure 1: Dynamics of the intestinal microbiota composition during the first 24 h after birth. (A) Image illustrating the study outline; one mouse from each one litter was analyzed for each individual time point (hours). **(B)** PCoA based on Bray-Curtis dissimilarity illustrating the small intestinal (SI) and colonic (C) microbial community structure along PC1 and PC2 during the first 24 hours (n=10 for 0-18 hours, n=6 for 24 hours, also for subsequent analyses in this figure). **(C and D)** Richness (observed species) of the **(C)** small intestinal ($p<0.0001$, linear regression $R^2=0.3548$) and **(D)** colonic ($p<0.0001$, linear regression $R^2=0.5360$) microbiota during the first 24 hours after birth, mean and SD. **(E-F)** Changes in the relative abundance of the 10 most abundant genera for both the small intestine **(E)** and colon **(F)** during the first 24 h after birth. Source data are provided as a Source Data file.

Supplementary Figure 2

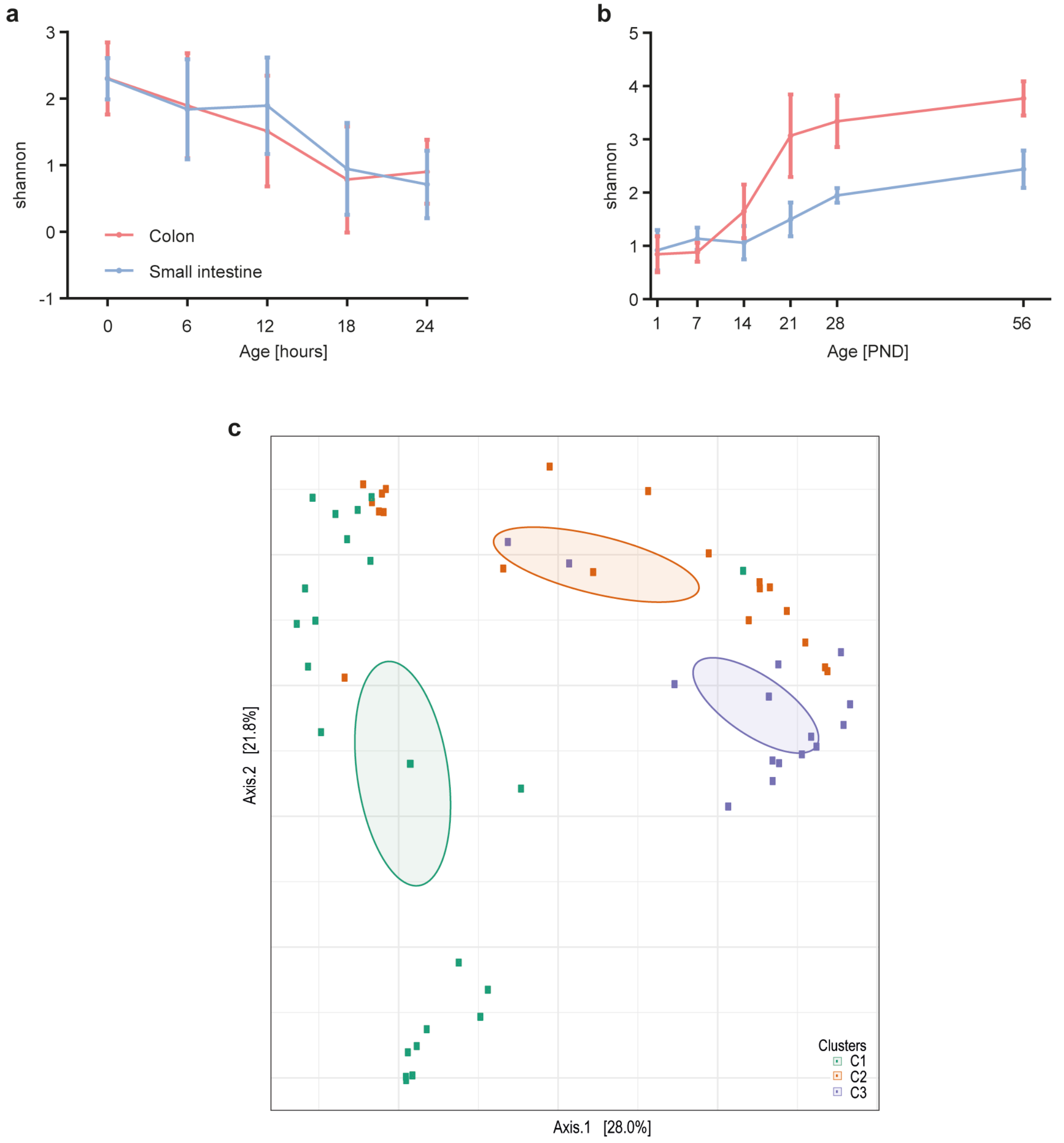

Supplementary Figure 2: Postnatal dynamics in microbial diversity. (A-B) Shannon index for the small intestinal (blue) and colonic (red) microbiota **(A)** between 0-24 h after birth (n=10 for 0-18 hours, n=6 for 24 hours) and **(B)** between 1-56 days (PND1-56) after birth (n=5 per group), , mean and SD **(C)** Three DMM-clustering profiles confirming the distinct microbial community structures of mainly small intestine and colon PND1-14 (C1, green symbols), small intestine PND21-56 (C2, orange symbols) and colon PND21-56 (C3, blue symbols) (p<0.001, Permanova). The ellipses depict the 95% confidence interval. Source data are provided as a Source Data file.

Supplementary Figure 3

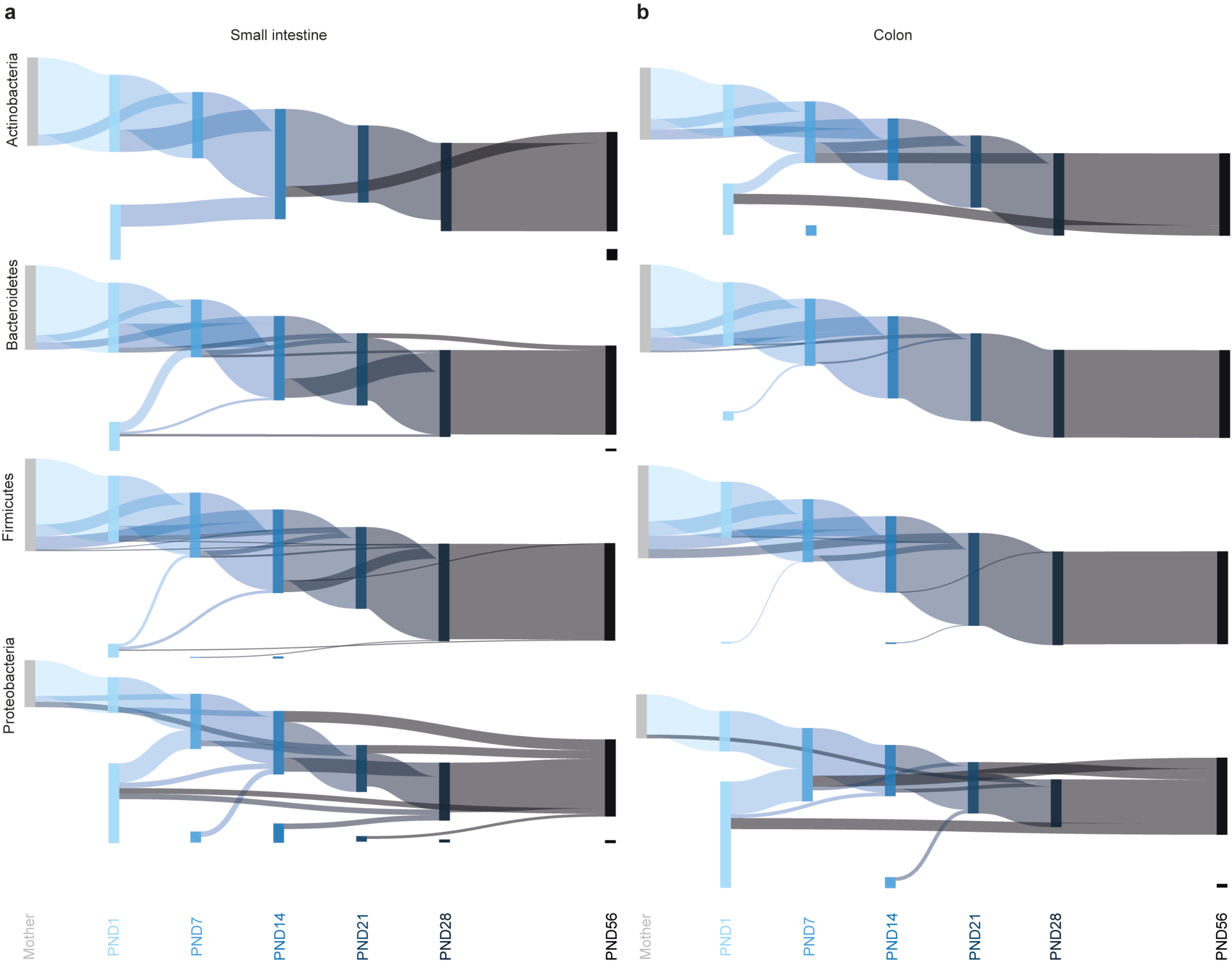

Supplementary Figure 3: Sankey plot tracking OTUs between PND1-PND56. (A and B) Sankey plot illustrating the origin and transmission of OTUs between mother and offsprings at the indicated time points after birth (postnatal day, PND). All OTUs shared by at least by 20 percent of the population in small intestinal **(A)** and colonic **(B)** microbiota were tracked and are shown individually for the four major phyla. The rectangle height indicates the relative number of OTUs (number of observed OTUs at a given time-point divided by the total number of OTUs within a phylum across all time-points) and the rectangle color reflects the age of the animals. Light grey rectangles represent the fecal microbiota of maternal animals. Lines represent the transfer of OTUs between different time points; the thickness of the line is indicative of the number of OTUs transferred.

Supplementary Figure 4

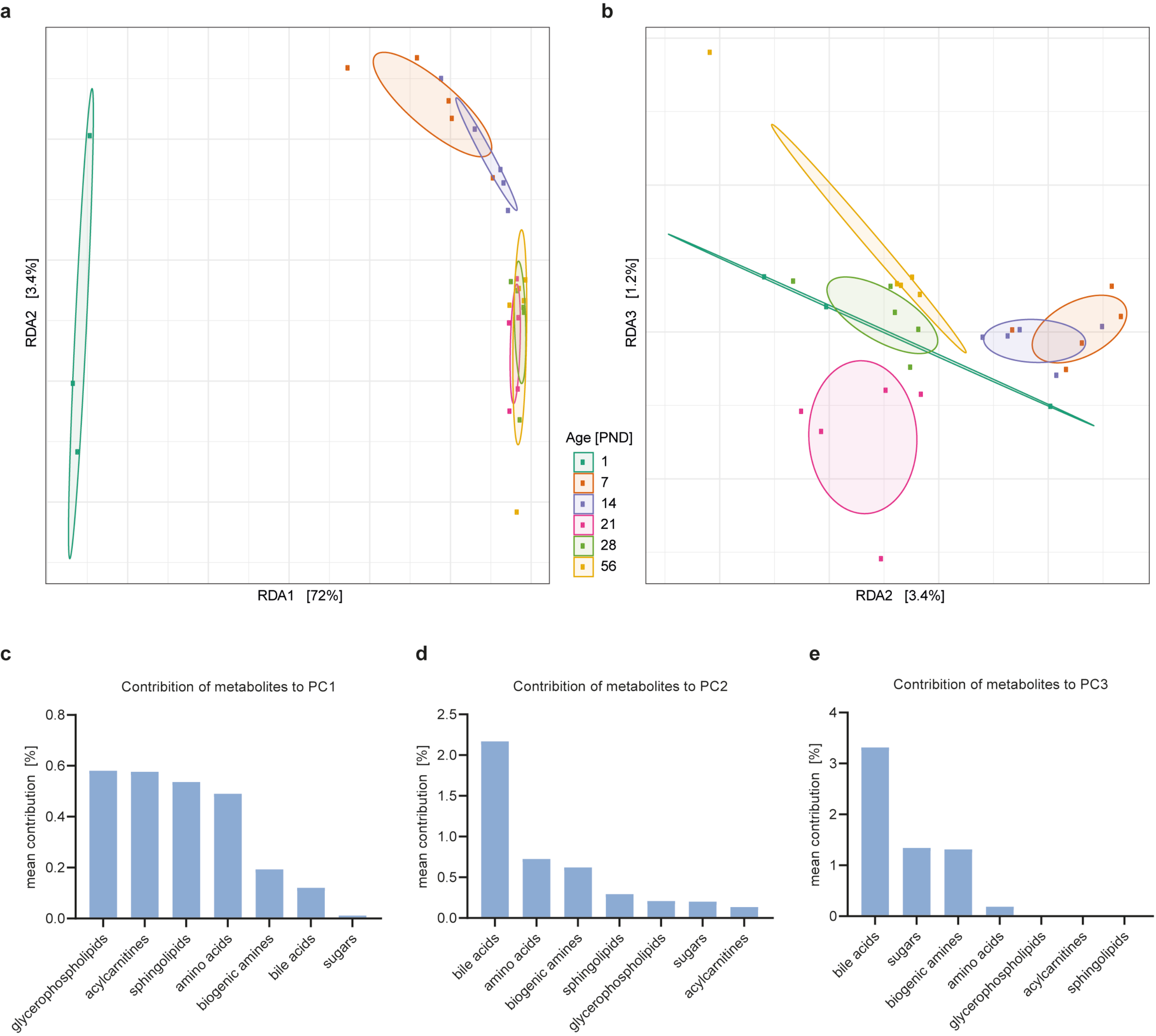

Supplementary Figure 4: The contribution of metabolic groups to age-dependent shifts in the metabolome. (A and B) Age-constrained ordination using redundancy analysis (RDA) of metabolomic groups colored according to age depicting **(A)** RDA1 and RDA2 and **(B)** RDA2 and RDA3 (n=5 for PND7-56 and n=3 for PND1). **(C-E)** Mean contribution of individual metabolic groups to explain the age-dependent variance of **(C)** RDA1, **(D)** RDA2 and **(E)** RDA3. Source data are provided as a Source Data file.

Supplementary Figure 5

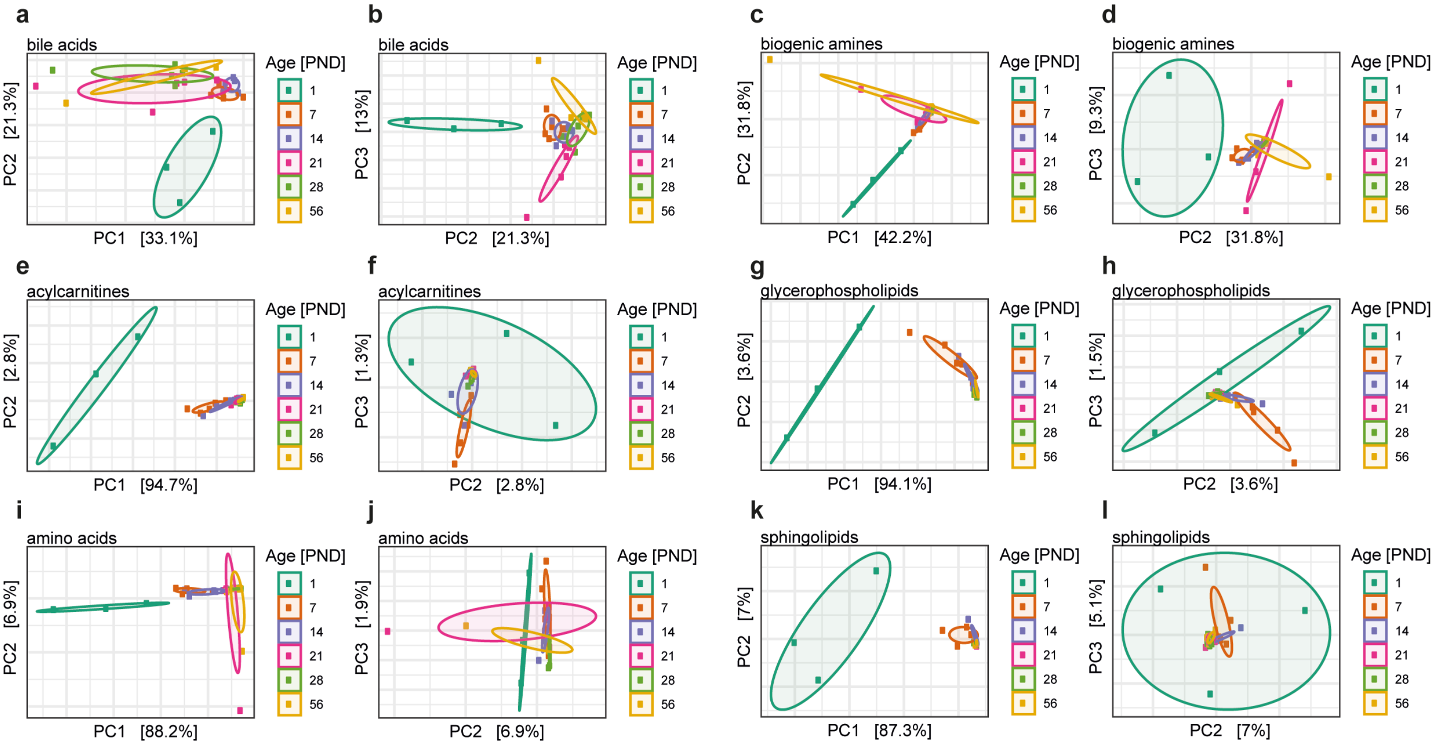

Supplementary Figure 5: Compositional changes of metabolic groups during the postnatal period. (A-L) Principal Component Analysis (PCA) illustrating changes in the composition of the indicated metabolic groups **(A and B)** bile acids, **(C and D)** biogenic amines, **(E and F)** acylcarnitines, **(G and H)** glycerophospholipids, **(I and J)** amino acids and **(K and L)** sphingolipids between PND 1 and 56. For each metabolic group, PC1 and PC2 **(A, C, E, G, I and K)** as well as PC2 and PC3 **(B, D, F, H, J, L)** are shown. Please note that since only the total sugar concentration was measured (single value/sample) we could not include sugars as a metabolic group. Due to the different number of metabolites in each group, the PCA's are not suitable for direct comparisons; see therefore Fig.2 and Fig. S4.

Supplementary Figure 6

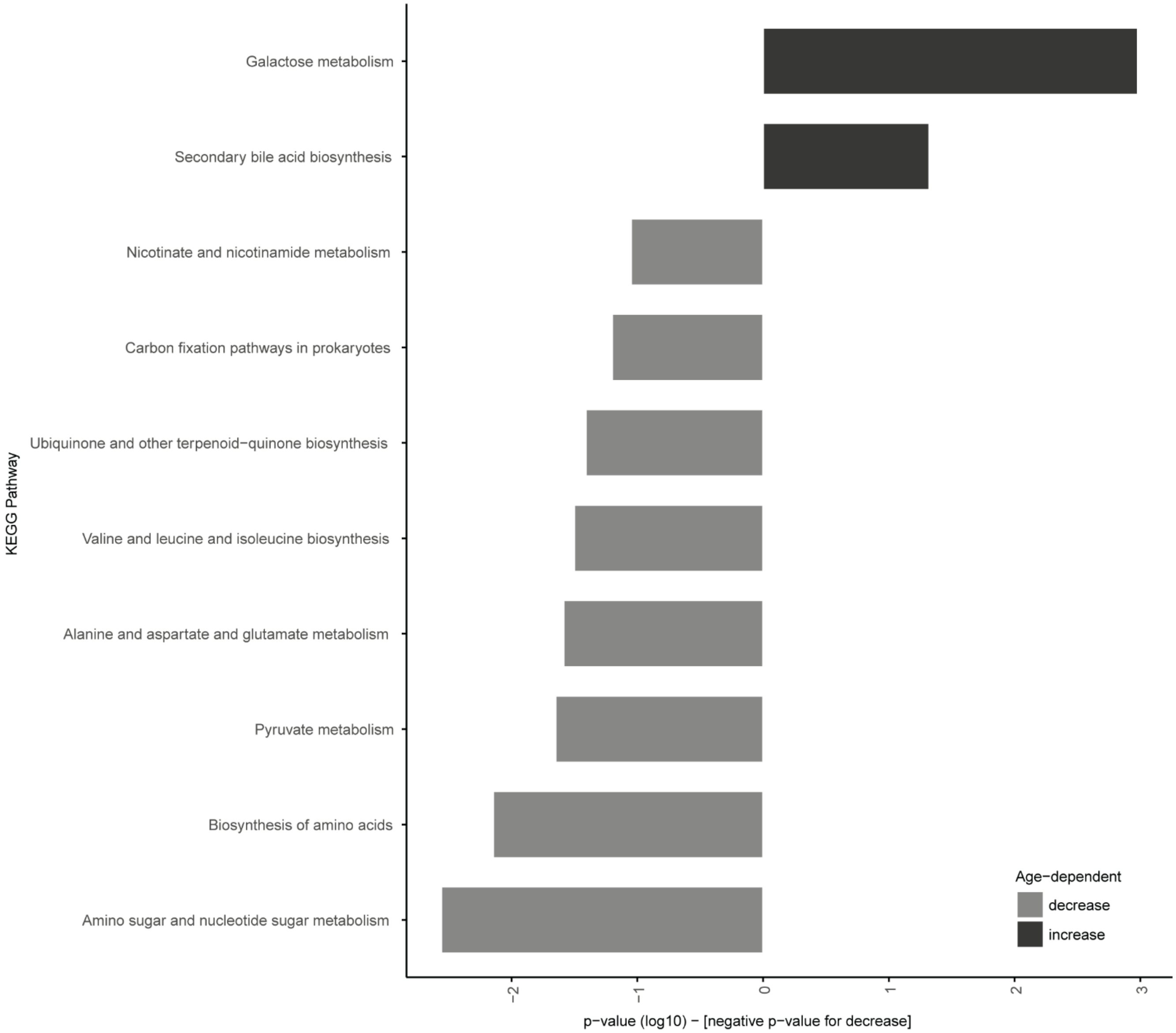

Supplementary Figure 6: Age-dependent metagenomic enrichment analyses. Mouse-PICRUSt based prediction of metagenomic information functionally annotated using the Kyoto Encyclopedia of Genes and Genomes (KEGG) database. Enrichment analysis of the age-dependent increases and decreases with the corresponding p-values for the top 10 differentiatially expressed KEGG pathways (n=5 per PND, PND1 vs. PND56, Global Test, corrected for multiple-testing using the Benjamini and Hochberg's False-Discovery Rate (FDR)).

Supplementary Figure 7

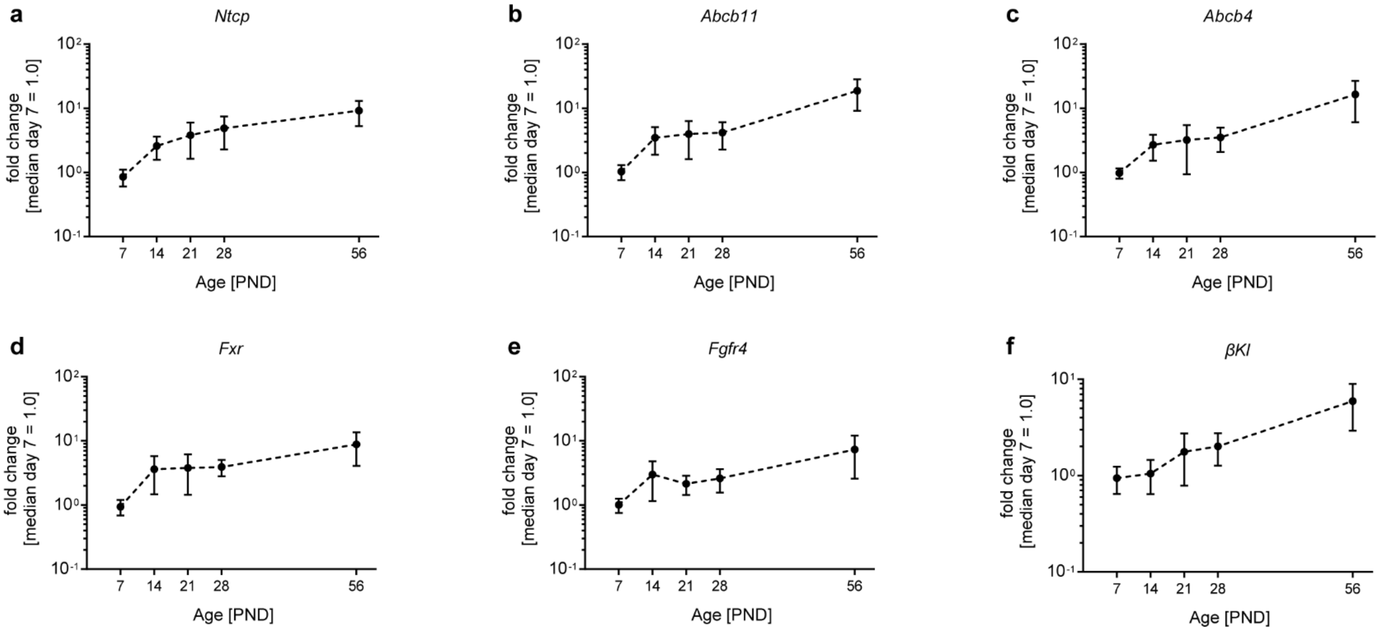

Supplementary Figure 7: Maturation of the enterohepatic cycle. (A-F) Expression analysis by RT-PCR for key factors in bile acid transport and signaling such as **(A)** the sodium/bile acid co-transporter Na^+-taurocholate cotransporting polypeptide (Ntcp), **(B)** the bile salt export pump ATP-binding cassette, sub-family B member (Abcb)11, **(C)** the lipid transport molecule ATP-binding cassette, sub-family B member (Abcb)4, **(D)** the bile acid receptor farnesoid X receptor (Fxr), **(E)** the fibroblast growth factor receptor (Fgfr)4, and **(F)** the Fgfr associated protein b-Klotho (bKl) in total liver tissue of mice at the indicated age (n = 5 per group, mean and SD). Source data are provided as a Source Data file.

Supplementary Figure 8

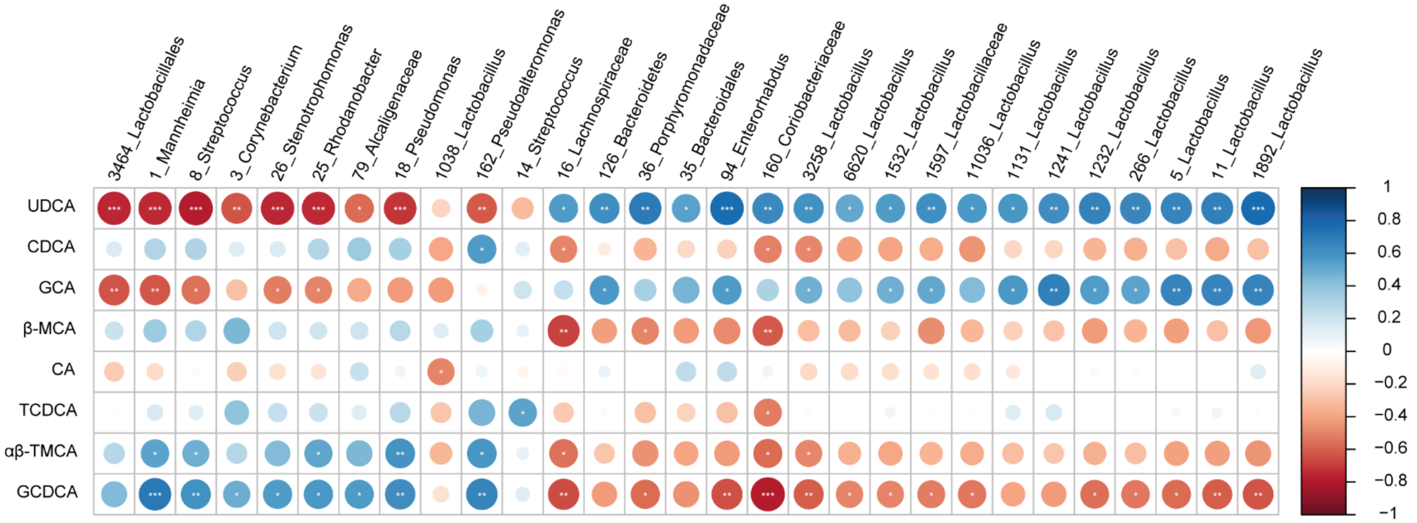

Supplementary Figure 8: Correlation heatmap of primary bile acids and individual OTUs. Correlation heatmap based on the rCCA with significant pairs of small intestinal OTUs/bile acids (Spearman, *p<0.05, **p<0.01, ***p<0.001).

Supplementary Figure 9

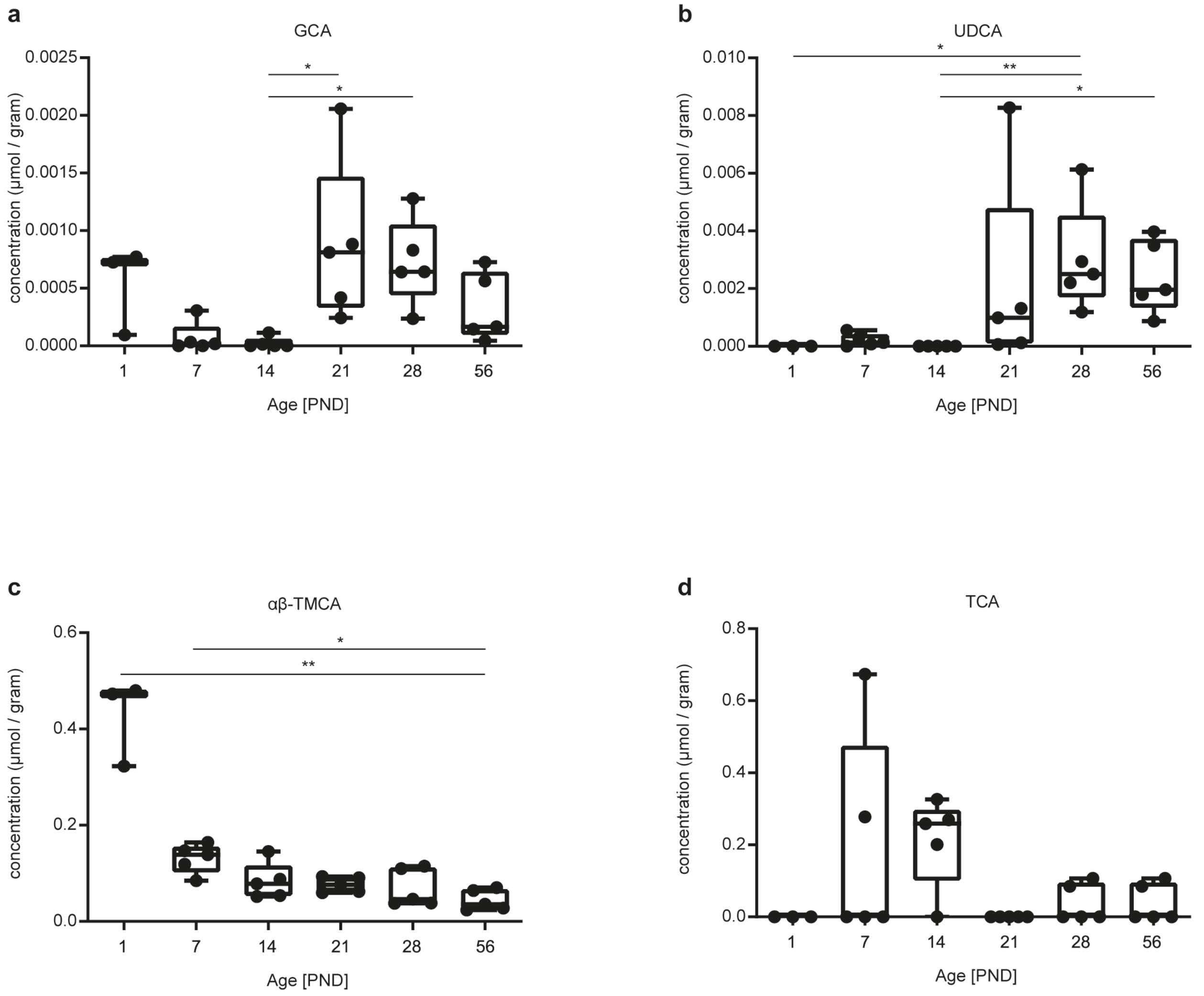

Supplementary Figure 9: Changes in bile acids according to age. A-D, Concentration [µmol/gram] of the four primary bile acids **(A)** UDCA, **(B)** αβ-TMCA**, (C)** GCA, and **(D)** TCA in total liver tissue (n=5 for PND7-56, n=3 for PND1, (Kruskal-Wallis test to controls with Dunn's post-test and correction for multiple comparisons , box represents IQR with median, whiskers represent minimum and maximum values; *, GCA: for PND14 vs PND21 p=0.0181, for PND14 vs PND28 p=0.0349. */**, UDCA: for PND1 vs PND28 p=0.0388, for PND14 vs PND28 p=0.0075, for PND14 vs PND56 p=0.0177. */**, αβ-TMCA: for PND1 vs PND56 p=0.0063, for PND7 vs PND56 p=0.0407, two-sided). Source data are provided as a Source Data file.

Supplementary Figure 10

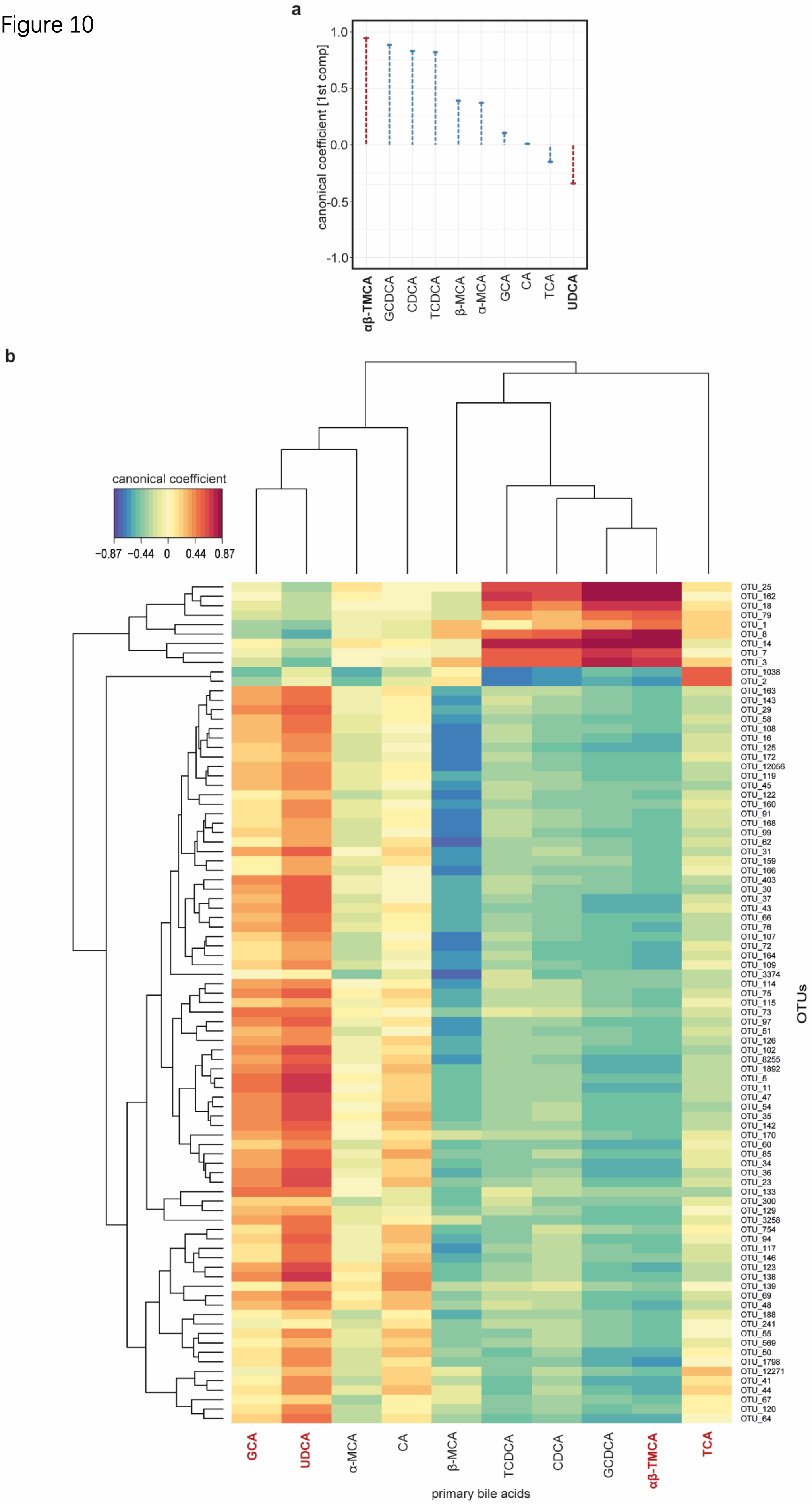

Supplementary Figure 10: Enterohepatic relationship between primary bile acids and colonic OTUs. **(A)** Coefficients of the regularized canonical correlation analyses (rCCA) indicating the relationship between primary bile acids and colonic OTUs on the first component (selected bile acids are highlighted in bold). **(E)** Correlation heatmap based on the coefficients of the rCCA between hepatic primary bile acid concentrations and relative abundances of colonic OTUs indicated a strong effect (red) of GCA, UDCA, TMCA and TCA.

Supplementary Figure 11

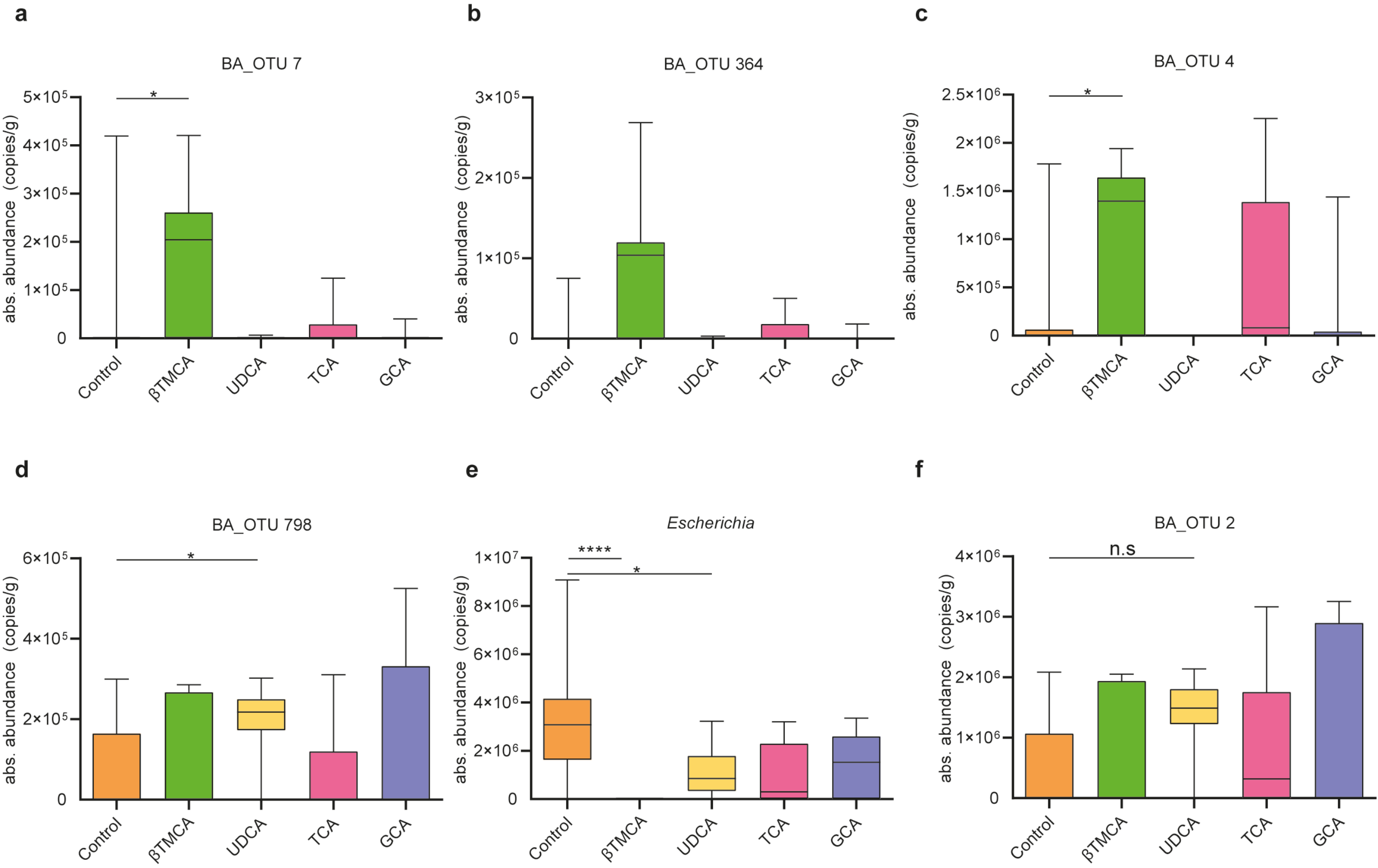

Supplementary Figure 11: Impact of orally administered bile acids on the absolute abundance of lactobacilli in the small intestinal microbiota. (A-F) Absolute abundances of the dominant lactobacillus OTUs **(A)** BA_OTU 7, **(B)** BA_OTU 364, **(C)** BA_OTU 4, **(D)** BA_OTU 798, **(E)** Escherichia and **(F)** BA_OTU 2 following oral administration of the indicated bile acids calculated based on the results of a quantitative analysis of the total 16S rDNA gene copy number (Kruskal-Wallis test to controls with Dunn's post-test and correction for multiple comparisons, box represents IQR with median, whiskers represent minimum and maximum values; *, BA_OTU7: for βTMCA vs Control $p=0.0180$. *, BA_OTU4: for βTMCA vs Control $p=0.0223$. *, BA_OTU798: for UDCA vs Control $p=0.0118$. */****, *Escherichia*: for βTMCA vs Control $p<0.0001$, for UDCA vs Control $p=0.0477$, two-sided. N=14 for UDCA and Control, n=11 for GCA, n=10 for TCA, n=7 for βTMCA).

Supplementary Figure 12

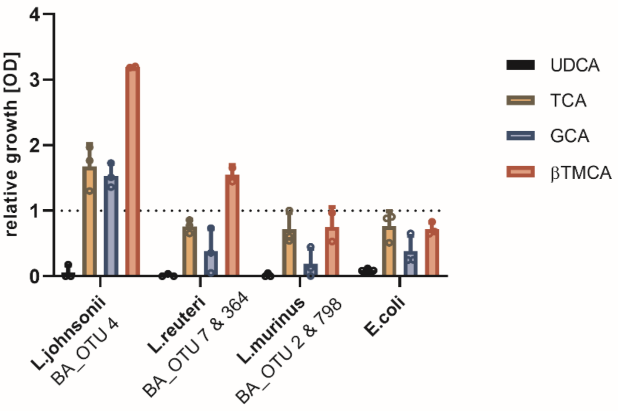

Supplementary Figure 12: Direct influence of bile acids on bacterial survival and growth. Influence of UDCA, TCA, GCA and bTMCA at a concentration of 1% on the anaerobic growth of representative murine lactobacillus and *E. coli* isolates as illustrated by the relative growth in the presence/absence of bile acid supplementation over 24 h (ΔOD_{570} in medium with 1% bile acid/ΔOD_{570} in medium with 0% bile acid), mean and SD, n=3 per group. Source data are provided as a Source Data file.
