# Supplementary material for: Bile acids drive the newborn’s gut microbiota maturation
Source: Nat Commun. 2020 Jul 23;11:3692. doi: 10.1038/s41467-020-17183-8 (PMC7378201; doi:10.1038/s41467-020-17183-8)
Supplement: Supplementary file 3 — Reporting Summary [file 41467_2020_17183_MOESM3_ESM.pdf]

naturereseach

Corresponding author(s): Mathias Hornef, John Penders

Last updated by author(s): 23.11.2019

Reporting Summary

Nature Research wishes to improve the reproducibility of the work that we publish. This form provides structure for consistency and transparency in reporting. For further information on Nature Research policies, see Authors & Referees and the Editorial Policy Checklist .

Statistics

For all statistical analyses, confirm that the following items are present in the figure legend, table legend, main text, or Methods section.

n/a	Confirmed	
☐	☒	The exact sample size (*n*) for each experimental group/condition, given as a discrete number and unit of measurement
☐	☒	A statement on whether measurements were taken from distinct samples or whether the same sample was measured repeatedly
☐	☒	The statistical test(s) used AND whether they are one- or two-sided<br>*Only common tests should be described solely by name; describe more complex techniques in the Methods section.*
☐	☒	A description of all covariates tested
☐	☒	A description of any assumptions or corrections, such as tests of normality and adjustment for multiple comparisons
☐	☒	A full description of the statistical parameters including central tendency (e.g. means) or other basic estimates (e.g. regression coefficient) AND variation (e.g. standard deviation) or associated estimates of uncertainty (e.g. confidence intervals)
☐	☒	For null hypothesis testing, the test statistic (e.g. *F*, *t*, *r*) with confidence intervals, effect sizes, degrees of freedom and *P* value noted<br>*Give P values as exact values whenever suitable.*
☒	☐	For Bayesian analysis, information on the choice of priors and Markov chain Monte Carlo settings
☒	☐	For hierarchical and complex designs, identification of the appropriate level for tests and full reporting of outcomes
☐	☒	Estimates of effect sizes (e.g. Cohen's *d*, Pearson's *r*), indicating how they were calculated

Our web collection on statistics for biologists contains articles on many of the points above.

Software and code

Policy information about availability of computer code

Data collection	Sequencing was performed on a Illumina MiSeq apparatus (M00403R). Metabolic profiling was performed on an ABI Sciex API5500 Q-TRAP mass spectrometer and concentrations were calculated by the integrated MetIDQ Software.
Data analysis	For microbiota analysis, data demultiplexing, length and quality filtering, pairing of reads and clustering of reads into Operational Taxonomic Units (OTUs) at 97% sequence identity was performed using the online Integrated Microbial Next Generation Sequencing (IMNGS, www.imngs.org) platform using default settings (Lagkouvardos et al., 2016a). Removal of primers and technical reads resulted in fragments of approximately 250 bases. Sequencing was performed from both the 3' and 5' side resulting in sufficient resolution. IMNGS is a UPARSE based analysis pipeline (Edgar et al., 2013). Pairing, quality filtering and OTU clustering (97% identity) was done by USEARCH 8.0 (Edgar et al., 2010). The analysis was based on OTUs rather than amplicon sequence variants (ASVs) since we aimed at aggregating taxa at a higher level and wanted to avoid overestimation of prokaryotic diversity due to Intragenomic heterogeneity of 16S rRNA genes (Sun et al., 2013). Chimera filtering was performed by UCHIME (with RDP set 15 as a reference database (Edgar et al., 2011). Taxonomic classification was done by RDP classifier version 2.11 training set 15.8 Sequence alignment was performed by MUSCLE and treeing by Fasttree (Edgar et al., 2004; Price et al., 2010). A total of 21,372,397 V4 reads were generated over two runs. After trimming, quality filtering, removal of potential chimeric reads, de-multiplexing and removal of low abundant operational taxonomic units (OTUs), 15,692,587 sequences belonging to 478 OTUs were retained for downstream analysis. Negative controls were evaluated based on their number of sequences and composition compared to other samples. We used for each batch, sampling blank controls, DNA blank extraction controls and no-template amplification controls, and monitored the lack of contaminant bacterial DNA load herein by a gel-based principle. Moreover, we compared the acquired OTUs composition from the negative controls to our low abundant microbial samples to ensure that our findings were not driven by potential contaminant taxa. Subsequently, samples of the negative controls and with low sequencing depth (less than 6,749 sequences/sample) were excluded from subsequent analysis. For the remaining samples, the number of sequences per sample ranged from 6,749 to 239,395 (median 87,227).<br>Data normalization, diversity, taxonomical binning and group comparisons were performed using the Rhea package version 1.6 (Lagkouvardos et al., 2017). In order to not discard informative data, normalization in Rhea was performed by dividing OTU counts per sample for their total count (sample depth) followed by multiplying all of the obtained relative abundance for the lowest sample depth (6,749 reads/sample). Alpha- (observed species and Shannon index) and the generalized Unifrac beta-diversity index were calculated

using the Rhea package (Chen et al., 2012). Additional beta-diversity indices (weighted Unifrac, unweighted Unifrac, and Bray-Curtis distance) were calculated using the R package (version 3.6.1.) Phyloseq package version 1.30.0 (McMurdie et al., 2012). Ordination of samples according to their microbial composition expressed as Hellinger transformed genus abundance data or beta-diversity indices was visualized using Principal Component Analysis (PCA) and Principal Coordinate Analysis (PCoA), respectively. All ordinations were constructed using the R package Phyloseq and included 95% confidence ellipses. Dirichlet multinomial mixtures models (DMM) were used to calculate genus-level enterotypes (Holmes et al., 2012). When including samples from all time-points, Laplace approximation revealed an optimal number of three clusters.

Smoothing of the kinetic for dominant taxa (Fig. 1e and f as well as Supplementary Figure 1d and e) was generated using the geom_smooth function of the ggplot package 3.2.1. with default settings. The lines reflect the mean values of the relative abundance. The appearance and disappearance of OTUs of the dominant phyla (Actinobacteria, Proteobacteria, Firmicutes, Bacteroidetes) with age was visualized in Sankey-plots (SankeyMATIC.com). For readability of Sankey-plots, only OTUs present in >10% of all samples per timepoint and with a prevalence of >20% in the entire dataset, were included. Ecosystem specific functional metagenome predictions were created by the novel PICRUSt-iMGMC workflow (using PICRUSt version 1.1.3) with the de novo picked OTUs and using mouse metagenome-assembled genomes linked to 16S rRNA genes (Lesker et al., 2019). The derived KEGG orthologs were mapped into multiple pathways or modules. Differentially changed KEGG modules were identified using the pathways enrichment analyses from MicrobiomeAnalyst with default settings (Stewart et al., 2018b). The identification of lactobacillus-related OTUs were performed using EZbiocloud and used to construct a phylogentic tree of lactobacilli by MEGA7 (MUSCLE) for alignment and iTOL v4 for the final annotations (itol.embl.de) with default settings.

Regularized canonical correlation analyses (rCCA) were performed (Mixomics package 6.10.8) (65) to unravel specific correlations between bile acids and OTUs with a minimal presence of 20% in all samples. Samples were excluded from the microbiota-data if they were not measured in the bile acid analyses (i.e. PND1 of litter 1 & 2). Prior to rCCA a hyperbolic sine transformation was used on OTU-counts and a log-transformation for the bile acids. For the estimation of regularization (penalization) parameters $\lambda 1$ and $\lambda 2$, the cross-validation procedure (CV) method was used. We used a $\lambda 1 = 0.0001$, $\lambda 2 = 1$ with a CV-score = 0.4779644 and 2 components. OTUs with a correlation between -0.3 and 0.3 on the first 2 components were filtered out to optimize the rCCA.

The Spearman's rank correlation coefficient was calculated between bile acids (weight corrected) and bacterial genera (relative abundances) with a minimal presence of 20% in all samples. Benjamini & Hochberg FDR correction was performed to correct for multiple testing (p>0.05). For the heatmap only significant correlations with adjusted p-value of <0.05 are shown and sorted according to their PCA loading scores.

For manuscripts utilizing custom algorithms or software that are central to the research but not yet described in published literature, software must be made available to editors/reviewers. We strongly encourage code deposition in a community repository (e.g. GitHub). See the Nature Research guidelines for submitting code & software for further information.

Data

Policy information about availability of data

All manuscripts must include a data availability statement. This statement should provide the following information, where applicable:

- Accession codes, unique identifiers, or web links for publicly available datasets
- A list of figures that have associated raw data
- A description of any restrictions on data availability

All data from this study are publicly available. V4 16S rDNA bacterial sequences have been submitted to the Qiita (https://qiita.ucsd.edu/study/description/10719) and ENA (https://www.ebi.ac.uk/ena/data/view/PRJEB33959) databases under accession No. 10719 and ERP116798 respectively. The matbolomic data are available at the NIH Common Fund's National Metabolomics Data Repository (NMDR) website, the Metabolomics Workbench, https://www.metabolomicsworkbench.org (http://dx.doi.org/10.21228/M8N397) where it has been assigned the Study ID ST001388 and the Project ID (PR000952). The data can be accessed directly via it's Project DOI: 10.21228/M8N397. This work is supported by NIH grant U2C-DK119886.

Field-specific reporting

Please select the one below that is the best fit for your research. If you are not sure, read the appropriate sections before making your selection.

☒ Life sciences ☐ Behavioural & social sciences ☐ Ecological, evolutionary & environmental sciences

For a reference copy of the document with all sections, see nature.com/documents/nr-reporting-summary-flat.pdf

Life sciences study design

All studies must disclose on these points even when the disclosure is negative.

Sample size	No sample size calculations were performed. Sample sizes were determined to be adequate based on the magnitude of measurable differences between groups as based upon samples sizes published in similar studies. All groups contained animals from more of one litter to avoid inter-litter variation effects.
Data exclusions	In some occasions, bacterial DNA did not yield sufficient DNA 16S amplicon or sequencing reads. No other pre-established inclusion/exclusion criteria were employed.
Replication	n/a (see below)
Randomization	Tissue samples from the indicated different time points after birth (0, 6, 12, 18, 24 hours or 1, 7, 14, 21, 28, 56 days) were each obtained sequentially from one litter housed in a single cage with one dam. All age groups contained animals from more of one litter to avoid inter-litter variation effects. Mouse litters were randomly assigned to experimental groups for bile acid supplementation.

Blinding	No blinded analyses were performed.

Reporting for specific materials, systems and methods

We require information from authors about some types of materials, experimental systems and methods used in many studies. Here, indicate whether each material, system or method listed is relevant to your study. If you are not sure if a list item applies to your research, read the appropriate section before selecting a response.

Materials & experimental systems

n/a	Involved in the study
☒	☐ Antibodies
☒	☐ Eukaryotic cell lines
☒	☐ Palaeontology
☐	☒ Animals and other organisms
☒	☐ Human research participants
☒	☐ Clinical data

Methods

n/a	Involved in the study
☒	☐ ChIP-seq
☒	☐ Flow cytometry
☒	☐ MRI-based neuroimaging

Animals and other organisms

Policy information about studies involving animals; ARRIVE guidelines recommended for reporting animal research

Laboratory animals	C57BL6/J wildtype mice (mus musculus) of both sexes raised under special pathogen-free (SPF) conditions were used at the indicated age in this study. All animal experiments were performed in compliance with the German animal protection law (TierSchG) and approved by the local animal welfare committees.
Wild animals	n/a
Field-collected samples	n/a
Ethics oversight	The local animal welfare committees, namely the Landesamt für Natur, Umwelt und Verbraucherschutz, North Rhine Westfalia (84-02.04.2016.A207 and 84-02.04.2015.A293) approved the performed experiments.

Note that full information on the approval of the study protocol must also be provided in the manuscript.
